# Cross-Linked Polyimide/ZIF-8 Mixed-Matrix Membranes by In Situ Formation of ZIF-8: Effect of Cross-Linking on Their Propylene/Propane Separation

**DOI:** 10.3390/membranes12100964

**Published:** 2022-10-01

**Authors:** Sunghwan Park, Hae-Kwon Jeong

**Affiliations:** 1 School of Energy Materials & Chemical Engineering, Kyungpook National University, Sangju-si 37224, Korea; 2 Department of Advanced Science and Technology Convergence, Kyungpook National University, Sangju-si 37224, Korea; 3 Artie McFerrin Department of Chemical Engineering, Texas A&M University, 3122 TAMU, College Station, TX 77843-3122, USA; 4 Department of Materials Science and Engineering, Texas A&M University, 3122 TAMU, College Station, TX 77843-3122, USA

**Keywords:** mixed-matrix membrane, zeolitic imidazolate framework, polymer cross-linking, in situ growth, propylene/propane separation

## Abstract

Despite their potential for the scalable production of mixed-matrix membranes (MMMs), the MMMs prepared by the polymer-modification-enabled in situ metal–organic framework formation (PMMOF) process showed a considerable reduction in gas permeability as the filler loading increased. It was hypothesized that a correlation existed between the decrease in permeability and the change in the properties of the polymer, such as free volume and chain flexibility, upon in situ MOF formation. Herein, we aim to address the permeability reduction by using a cross-linked polyimide (6FDA-DAM:DABA (3:2)). It was found the degree of cross-linking affected not only the properties of the polymer, but also the in situ formation of the ZIF-8 filler particles in the cross-linked polymer. The proper degree of cross-linking resulted in suppressing C_3_H_6_ permeability reduction, suggesting a possible strategy to overcome the issue of PMMOF. The swelling of the polymer followed by chain rearrangement during the PMMOF, as well as the structural rigidity of the polymer, were found to be critical in mitigating permeability reduction.

## 1. Introduction

Mixed-matrix membranes (MMMs) are promising alternatives to the current polymer gas separation membranes, whose performances are limited by the trade-off between permeability and selectivity [1]. A number of studies have shown that incorporating highly permeable and/or selective molecular sieve fillers in polymer matrices led to improved gas separation performances of the polymer membranes, overcoming their intrinsic limitations [2,3,4].

Despite their potentials [5,6,7], there have been only a few reports on MMMs in more scalable geometries, such as hollow fibers (i.e., mixed-matrix hollow fiber membranes with submicron selective skin layers) for large-scale applications [8]. This is due to several challenges of applying established hollow fiber spinning processes to mixed-matrix hollow fiber membrane (MMHFM) fabrication, often leading to a poor interface between the polymer matrix and fillers [9], filler agglomeration [10], several micron-thick selective skin layers [11], and others. It is extremely difficult to address the above-mentioned challenges when fillers need to be incorporated into the submicron selective skin layers of MMHFMs [11].

Recently, there have been several reports that fabricate MMMs using in situ filler growth to overcome the challenges of the conventional MMM processing technologies [12,13,14]. Among these, polymer-modification enabled in situ metal–organic framework formation (PMMOF) stands out because it was able to successfully address the above-mentioned issues associated with scalable MMM formation [14,15,16]. PMMOF decouples a polymer membrane fabrication step from a filler incorporation step by growing MOF fillers in situ in a modified-polyimide film. The resulting ZIF-8-containing MMMs exhibited excellent C3 separation compared with those MMMs using the conventional blending method. It is noted that due to its effective aperture size (i.e., 4.0~4.2 Å), ZIF-8 is known for excellent kinetic separation of the C_3_H_6_ and C_3_H_8_ mixture [17]. Furthermore, we demonstrated the first MMM module containing multi-stranded mixed-matrix hollow fiber membranes with submicron-thick selective skin layers by transforming a preformed module with PI-coated polyethersulfone hollow fibers using PMMOF [18].

Unfortunately, MMMs made by PMMOF showed lower gas permeabilities than those prepared by the conventional blending method [14,15]. The lower permeability was attributed possibly to the polymer densification/rigidification upon the in situ formation of fillers [14]. It is known that the gas permeability in the polymer decreases due to reduced polymer free volume and/or restricted chain mobility [19]. Polymer rigidification is expected to be more pronounced in MMMs prepared by PMMOF than in conventional MMMs. This is because in situ filler formation during PMMOF results in fillers with a much smaller in size (<100 nm) and enhanced compatibility with the polymer, thereby providing larger and more compatible polymer/filler interfaces, and consequently a more restricted polymer chain mobility [20]. Furthermore, the destructive and complicated nature of the PMMOF process likely led the modification (i.e., densification) of the polymer microstructure. Therefore, we hypothesize that polymers with more rigid structures and higher free volumes may reduce further rigidification/densification upon PMMOF, thereby mitigating the decrease in permeability.

Cross-linking polymer chains is an effective means to obtain a more rigid polymer structure. Cross-linking has been widely used in polymer gas separation membranes to enhance their resistance to plasticization under aggressive condensable gas conditions such as CO_2_, CH_4_, and C_3_H_6_ [21,22]. As a derivative of 4,4′-(Hexafluoroisopropylidene)diphthalic anhydride (6FDA)-base polyimides, 4,4’-(hexafluoroisopropylidene) diphthalic anhydride- diaminobenzoic acid 2,4,6-trimethyl-1,3-phenylenediamine (6FDA-DAM:DABA) is a thermally cross-linkable polymer that has been extensively studied for gas separations [23,24,25,26]. Sub-*T_g_* cross-linking of 6FDA-DAM:DABA (3:2) led to excellent plasticization resistance [23,25]. Furthermore, the nano-scale chain rearrangement upon cross-linking enhanced the gas permeabilities of the polymer several times [23].

Many MMMs made of a cross-linked polymer have been prepared by cross-linking after the fabrication of MMMs with uncross-linked polymers. However, as of yet, there has not been a report on the fabrication of MMMs using cross-linked polymers directly via in situ filler formation strategies. In this study, we first demonstrated the formation of ZIF-8-containing MMMs directly using cross-linked 6FDA-DAM:DABA (3:2) polymer films by PMMOF. The polymer films were cross-linked at different temperatures, resulting in different degrees of cross-linking. We investigated the effect of the degree of cross-linking on the in situ formation of ZIF-8 filler particles in the cross-linked polymer. Then, we tested the C_3_H_6_/C_3_H_8_ separation performances of the resulting MMMs. The results indicated that the degree of cross-linking played a key role in mitigating the C_3_H_6_ permeability and controlling the C_3_H_6_/C_3_H_8_ separation performances.

## 2. Experimental

### 2.1. Materials

4,4′-(hexafluoroisopropylidene) diphthalic anhydride- diaminobenzoic acid 2,4,6-trimethyl-1,3-phenylenediamine (6FDA-DAM:DABA) (3:2) with Mw of 223k and PDI of 2.37 was purchased from Akron Polymer Systems Inc. (Akron, OH, USA). Polymer of intrinsic microporosity-1 (PIM-1) was kindly provided by Hanyang University in Korea. Sodium formate (HCOONa, ≥99%), zinc nitrate hexahydrate (Zn(NO_3_)_2_·6H_2_O, 98%), and 2-methylimidazole (HmIm) (C_4_H_6_N_2_, 99%) were from Sigma-Aldrich (Burlington, MA, USA). Methanol (CH_3_OH, >99.8%), chloroform (CHCl_3_, >99.8%), and N,N-dimethylformamide (DMF) (C_3_H_7_NO, >99.8%) were obtained from Alfa Aesar (Ward Hill, MA, USA). All of the chemicals were used as-received, without further purification.

### 2.2. Preparation of Polymer Membranes

Polymer films were prepared by a drop casting method on porous α-alumina disks. The preparation of α-alumina disks is described elsewhere [27]. A 2 wt% 6FDA-DAM:DABA (3:2) solution was prepared by dissolving it in DMF. Then, 0.24 mL of the polymer solution was slowly dropped onto an α-alumina disk. Immediately after, the sample was placed in a vacuum oven, pre-heated at 150 °C, and dried for 1 day. As a reference, PIM-1 polymer films were prepared. A 2 wt% PIM-1 polymer solution in CHCl_3_ was casted onto an α-alumina disk in a solvent-saturated glove bag. For both samples, the polymer layers on the α-alumina disks were ~8 μm thick.

### 2.3. Cross-Linking of Polymer Membranes

The film samples of 6FDA-DAM:DABA (hereafter, PI) were thermally cross-linked at 370 °C and 420 °C, and were denoted as X-PI(370) and X-PI(420), respectively, for 120 min with a ramp rate of 10 °C min^−1^ under the argon flow of 200 cm^3^ min^−1^ in a tube furnace (Thermo Scientific, Waltham, MA, USA). Before heating, the reactor was purged with UHP argon for at least 1 h at room temperature. The gas flow rate was controlled using a mechanical flowmeter (Cole Palmer, Vernon Hills, IL, USA).

### 2.4. Preparation of MMMs by the PMMOF

The PMMOF process involves hydrolysis, ion exchange, ligand treatment, and imidization [14]. The cross-linked polymer films were hydrolyzed at different conditions depending on the degree of cross-linking. Here, 0.67 M and 3.33 M sodium formate solutions were prepared by dissolving 20 mmol and 100 mmol of sodium formate in 30 mL of DI water, respectively. X-PI(370) was hydrothermally hydrolyzed at 120 °C for 3 h in a Teflon-lined autoclave containing the 0.67 M sodium formate solution with the film vertically placed in a custom-made Teflon holder. X-PI(420) was similarly hydrolyzed in the 3.33 M sodium formate solution at 120 °C for 5 h. It is noted that the hydrolysis conditions were chosen to achieve similar degrees of hydrolysis (~70%) for both X-PIs, in order to achieve the maximum effect of the PMMOF while maintaining the structural integrity of the polymers, as reported in our previous study [14]. For the ion exchange, the hydrolyzed films were placed in zinc solutions of varying concentrations (20 mmol, 40 mmol, and 60 mmol of zinc nitrate hexahydrate in 40 mL of DI water) for 2 h. After briefly washing with methanol, the films were then immersed into a ligand solution (25 mmol of HmIm in 30 mL of methanol) and the reaction was carried out at 40 °C for 2 h. Afterward, the films were washed in flash methanol for 1 day at room temperature using a lab shaker. Finally, thermal imidization was performed at 250 °C for 3 h in a pre-heated convection oven. For comparison, PIM-1/ZIF-8 MMMs were prepared by PMMOF with a slight modification, as PIM-1 is not hydrolysable. The prepared PIM-1 films were immersed into ion exchange solutions with a zinc concentration of 20 mmol and 40 mmol. Followed by a brief washing step with methanol, the ligand treatment was conducted using the ligand solution that was prepared by dissolving 25 mmol of HmIm in 30 mL of methanol at 40 °C for 2 h. After washing with methanol at room temperature overnight, the samples were dried at 120 °C for 1 h.

### 2.5. Characterizations

The thermogravimetric analysis (TGA, Q50 TA instruments) was carried out at a temperature range of 25 °C to 700 °C, with a heating rate of 10 °C/min under air or argon flow of 50 cm^3^ min^−1^. Differential scanning calorimetry (DSC; Q20 TA instruments) was performed by ramping the temperature from 25 °C to 420 °C, at a rate of 5 °C/min under 100 cm^3^ min^−1^ of argon flow using Tzero aluminum hermetic pans. All of the DSC results were taken from the first scan to avoid thermal hysteresis of the polymer. Electron micrographs were taken using a scanning electron microscope (SEM; JEOL JSM-7500F, Tokyo, Japan) operated at an acceleration voltage of 5 keV with a working distance of 15 mm. Powder X-ray diffraction (PXRD; Rigaku Miniflex II) patterns were taken using Cu-Kα radiation (λ = 1.5406 Å) at a 2 *θ* range of 5–40°. Fourier transform infrared (FT-IR) spectra were taken using a spectrometer (Nicolet iS5 Thermo Scientific) equipped with an attenuated total reflectance (ATR, iD7) accessory at a wavenumber range of 4000–400 cm^−1^ with a resolution of 4 cm^−1^ and 16 scans.

### 2.6. Gas Permeation Measurements

The C3 gas permeation tests were conducted using the Wicke–Kallenbach technique using an equimolar binary C_3_H_6_ and C_3_H_8_ gas mixture at room temperature under 1 atm. Both the feed and argon sweep gases were supplied at a flow rate of 20 cm^3^ min^−1^. The permeation performances of the membranes were measured at steady states. Steady states were declared when the variation of the gas permeance reached less than 1% with 30 min intervals. The gas compositions on the permeate side were determined using gas chromatography (GC 7890A, Agilent, Santa Clara, CA, USA) equipped with a flame ionized detector (FID) and a HP-plot Q column.

## 3. Results and Discussion

### 3.1. Fabrication of Cross-Linked-PI/ZIF-8 MMMs by the PMMOF

**Figure 1:**
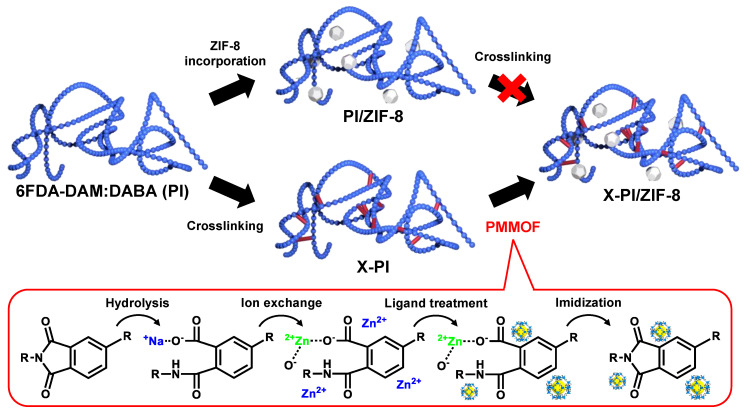
Schematic illustration of preparing cross-linked PI/ZIF-8 MMMs with two different routes: ZIF-8 incorporation either using the conventional blending method or the PMMOF followed by cross-linking (upper arrows) vs. cross-linking followed by PMMOF (see the four-step process in red box) (lower arrows). The red cross on the second upper arrow indicates that thermal cross-linking is not feasible because of the degradation of ZIF-8 fillers at cross-linking temperatures.

Figure 1 shows two different approaches to prepare MMMs with cross-linked 6FDA-DAM:DABA (3:2) polymer (X-PI) and ZIF-8 fillers. The first approach seems feasible using either the conventional blending methods or the PMMOF process. It involves the incorporation of ZIF-8 fillers in an uncross-linked polymer (PI) followed by cross-linking at a temperature below the decomposition temperature of ZIF-8 (i.e., *T_d_* of ZIF-8 ~300 °C and ~500 °C in air and inert gas, respectively) [28]. However, this approach poises a critical challenge, where the ZIF-8 structure can be compromised not only of thermal treatment at temperatures above 330 °C, but also by the presence of acidic DABA moieties (i.e., carboxyl groups) [23,29]. Furthermore, Lively et al. [29] reported the gelation of ZIF-8-containing 6FDA-DAM:DABA (4:1) dope solutions upon sonication, thereby fabricating 6FDA-DAM:DABA (4:1)/ZIF-8 MMMs with extra cautions [29]. Considering these challenges, we decided to use the second approach where the polymer is first cross-linked, followed by the in situ formation of ZIF-8 fillers inside the cross-linked polymer via the PMMOF process, as illustrated in Figure 1 (see the lower arrows). Cross-linked polymers are not easily dissolved in common organic solvents [23,30], which makes it difficult to prepare MMMs using the conventional blending methods. In contrast, PMMOF does not require dissolution of the polymers, because ZIF-8 fillers form inside the polymer-free volumes, resulting in it being much more effective at fabricating X-PI/ZIF-8 MMMs than the conventional blending methods.

### 3.2. Thermal Cross-Linking of Polymer

**Figure 2:**
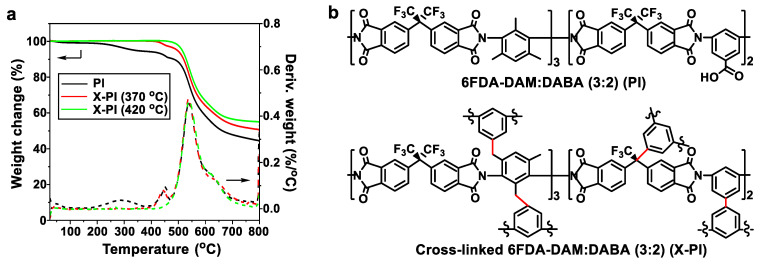
(**a**) TGA thermograms of free-standing 6FDA-DAM:DABA (3:2) (PI), X-PI (370), and X-PI (420), and (**b**) a possible chemical structure of the cross-linked PI in comparison with the chemical structure of PI.

Figure 2a presents the thermal decomposition behaviors of a pristine PI and two X-PIs treated at 370 °C and 420 °C, which are below and above the reported *T_g_* (~387 °C), respectively [23,26]. For PI, a minor weight loss was observed in the temperature span of ca. 400~465 °C, with a weight change of ~3.3 wt% (Figure 2a). This minor weight loss is attributed to the thermal decarboxylation and subsequent generation of phenyl radicals, consequently leading to decarboxylation-induced polymer cross-linking (Figure 2b) [23,31]. Following the minor weight loss, there was a major weight loss resulting from the degradation of polymer chain backbones in the temperature range of ca. 465~800 °C, with the additional weight loss of ~46 wt% (Figure 2a). The X-PI samples treated at 370 °C (hereafter, X-PI(370)) and at 420 °C (hereafter, X-PI(420)) showed ~2.9 wt% and ~0.4 wt% loss in the range of ca. 400~465 °C, respectively (Figure 2a). This indicates a partial loss of the carboxyl groups of the X-PI(370) and almost complete removal of the carboxyl groups of the X-PI(420).

To confirm cross-linking, the solubilities of free-standing PI and X-PI films were tested by immersing 10 mg of the samples in 2 mL of DMF at room temperature. As expected, the pristine PI films were immediately dissolved in DMF, whereas the X-PI films were swollen but preserved for at least one day, confirming a decrease in their solubilities upon cross-linking (Figure S1). The X-PI(370) was swollen more intensely and rapidly than the X-PI(420), likely due to its lower degree of cross-linking [32]. Figure S2 presents the X-ray diffraction patterns of the X-PI samples in comparison with those of the PI sample. As shown in the figure, the PI sample shows two broad peaks at 2*θ* of ~13.4° and ~15.5°, suggesting the presence of two inter-chain distances, ~6.6 Å and ~5.7 Å. Upon cross-linking, the intensity of the peak at ~13.4° increased, while that of the peak at ~15.5° decreased. This result is consistent with the previous report that the average inter-chain distance was enlarged upon cross-linking (i.e., the portion of the inter-chain distance of ~6.6 Å increased relative to that of ~5.7 Å), suggesting an increase in the polymer free-volume [23]. Furthermore, the *T_g_* of the X-PI samples increased from ~367 °C to ~381 °C and ~415 °C upon cross-linking at 370 °C and 420 °C, respectively (see Figure S3), indicating a significantly restricted polymer chain flexibility with the increase in the degree of cross-linking.

### 3.3. In Situ ZIF-8 Formation in Cross-Linked Polymers

**Figure 3:**
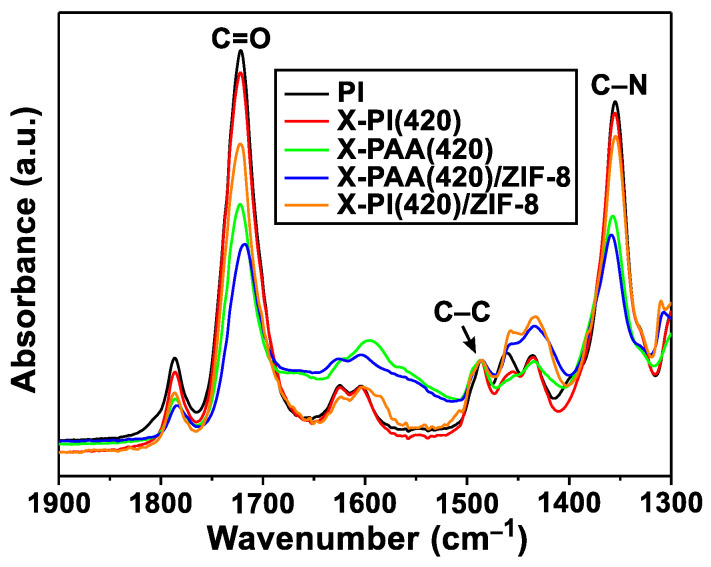
FT-IR spectra of the X-PI(420) sample at each polymer modification step in comparison with that of the pristine PI.

The in situ formation of ZIF-8 in X-PIs was performed by PMMOF, and involves four steps: hydrolysis, ion exchange, ligand treatment, and imidization (see the red box in Figure 1) [14]. First, the imide rings of the X-PI sample were cleaved via hydrolysis, turning it into cross-linked poly(amic acid) (X-PAA). It is noted that the less cross-linked X-PI(370) samples were more prone to hydrolysis than the more cross-linked X-PI(420), requiring milder hydrolysis to achieve a similar degree of imidization. Because of the similar degree of imidization, X-PI(370) showed a similar FT-IR spectrum as X-PI(420) (see Figure S4). As such, only the X-PI(420) samples are discussed in this section. Upon hydrolysis of the X-PI(420) sample, the intensities of the asymmetric C=O stretching (~1722 cm^−1^) and C-N stretching (1355~1359 cm^−1^) of the imide rings decreased compared with that of the C-C stretching of the benzene rings (~1486 cm^−1^) (Figure 3). Based on the ratio of the C-N and C-C stretching intensities, the degree of imidization of the resulting X-PAA(420) was estimated at ~70% [14]. Once ZIF-8 was formed in situ, X-PAA(420)/ZIF-8 was imidized, resulting in an increase in the normalized intensities of the asymmetric C=O and C-N stretching by that of the C-C stretching of X-PAA/ZIF-8 (Figure 3 and Figure S4). This indicates imide ring formation from the carboxylic salts of the X-PAA(420)/ZIF-8, thereby forming X-PI(420)/ZIF-8. The degree of imidization of the X-PI(420)/ZIF-8 increased to ~90% from ~60% of the X-PAA(420)/ZIF-8, which is comparable with that reported in our previous work [14].

**Figure 4:**
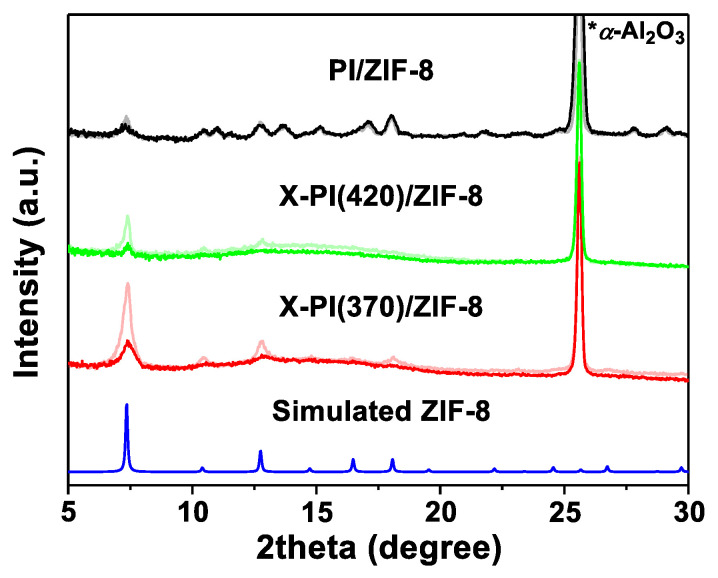
XRD patterns of X-PI(370)/ZIF-8, X-PI(420)/ZIF-8, and PI/ZIF-8. The overlapped lines in lighter colors are the diffraction patterns of the samples before acid treatment and presented for a comparison purpose.

**Figure 5:**
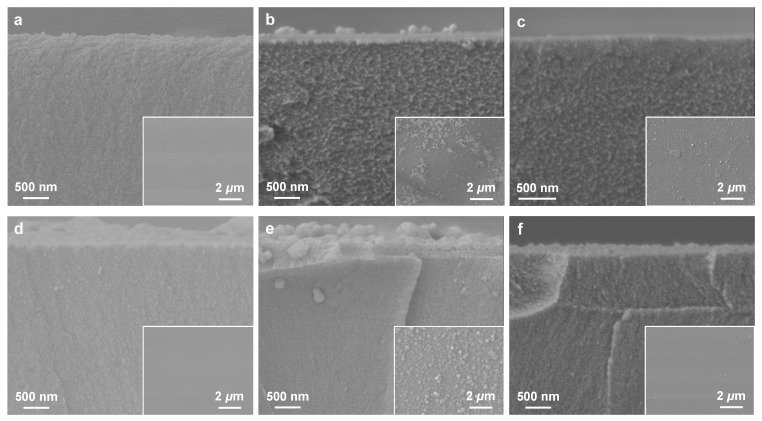
Cross-sectional SEM images of (**a**) X-PI(370), (**b**) as-prepared and (**c**) acid-treated X-PI(370)/ZIF-8, (**d**) X-PI(420), (**e**) as-prepared X-PI(420)/ZIF-8, and (**f**) acid-treated X-PI(420)/ZIF-8. The inset images are the corresponding top views.

To confirm the presence of in situ formed ZIF-8 inside the X-PIs, all surface-bound ZIF-8 particles were removed by gently wiping the top sample surface with a Kimwipe soaked with a diluted acid solution (i.e., 0.1 M of H_2_NO_3_). As shown in Figure 4, the XRD intensities of both X-PI(370)/ZIF-8 and X-PI(420)/ZIF-8 samples decreased after the acid treatment. The corresponding SEM images showed that the surface-bound ZIF-8 clusters were eliminated by the acid treatment (Figure 5). Nevertheless, ZIF-8 diffraction patterns remained (Figure 4), indicating that ZIF-8 particles were formed inside the X-PI films by PMMOF. In addition, the XRD showed that the (011) peak of X-PI(370)/ZIF-8 was stronger than that of X-PI(420)/ZIF-8, indicating that more ZIF-8 filler particles formed inside X-PI(370) than X-PI(420). On the other hand, the broader and much smaller (011) peak of the uncross-linked PI/ZIF-8, along with unidentified peaks, strongly suggested the crystallinity of ZIF-8 formed in situ inside the uncross-linked PI was compromised, likely due to the decomposition by the acidic DABA moieties of the polymer (Figure 4).

As seen in Figure 5a–c, X-PI(370)/ZIF-8 showed a rough cross-sectional surface compared with X-PI(370), which was consistent with the previous observation made in uncross-linked 6FDA-DAM/ZIF-8 MMMs [14]. In stark contrast, X-PI(420)/ZIF-8 showed a smooth cross-sectional surface comparable with X-PI(420) (Figure 5d–f), which might be attributed to the suppressed formation of ZIF-8, as observed in the XRD. The high degree of cross-linking likely impeded zinc uptake in the free volume by obstructing the swelling of the polymer [33], thereby leading to a smaller amount of ZIF particles forming than in X-PI(370)/ZIF-8. Under the same ion exchange conditions (i.e., 0.5 M zinc solution), the ZIF-8 loadings in X-PI(370)/ZIF-8 and X-PI(420)/ZIF-8 were estimated at ~8 wt% and ~3 wt%, respectively (a detailed analysis of the ZIF-8 loadings is presented in the paragraph below). As such, it was surmised that X-PI(370) was more prone to swelling due to the lower degree of cross-linking, enabling the in situ formation of a relatively large number of ZIF-8 particles, leading to the rough cross-sectional surface of X-PI(370)/ZIF-8.

**Figure 6:**
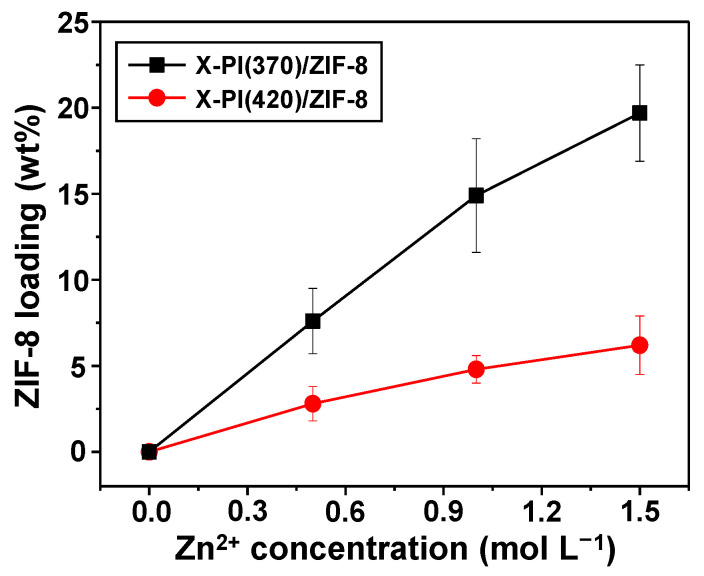
ZIF-8 loadings in X-PI/ZIF-8 MMMs as a function of the zinc concentrations of the ion exchange solutions.

As the zinc concentration of the ion exchange solution increased from 0.5 M to 1.0 M to 1.5 M, the ZIF-8 loading in X-PI increased continuously (Figure 6 and Table S1) [14]. The ZIF-8 loading was determined based on the residual weight of the corresponding free-standing MMM sample upon thermal oxidation (Figure S5). As shown in Figure 6, the X-PI(370)/ZIF-8 samples showed not only significantly more ZIF-8 loadings, but also a much sharper increase than X-PI(420)/ZIF-8. This is likely due to the favorable formation of ZIF-8 particles resulting from the lower degree of cross-linking of X-PI(370).

**Figure 7:**
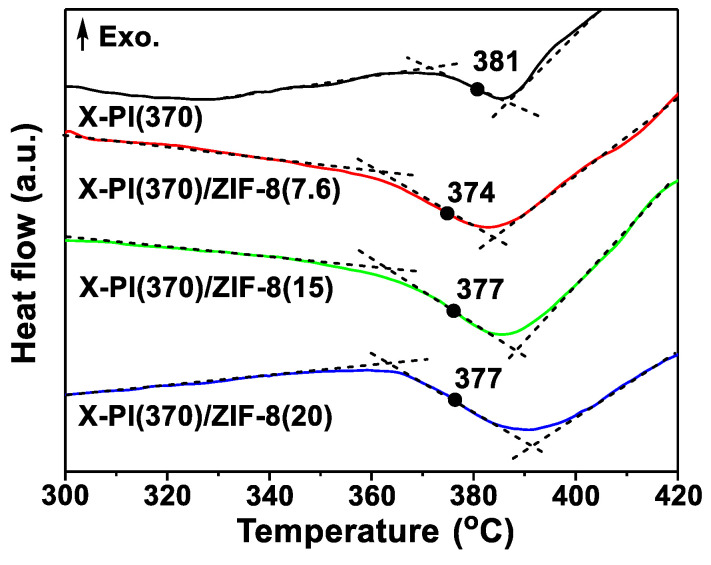
DSC thermograms of X-PI(370) and X-PI(370)/ZIF-8 MMMs. The numbers in the parentheses are the ZIF-8 loadings.

To check the chain flexibility of the X-PI/ZIF-8 MMMs, differential scanning calorimetry (DSC) experiments were performed. As shown in Figure 7, the *T_g_* of X-PI(370)/ZIF-8 decreased relative to that of X-PI(370), suggesting that the cross-linked polymer became less rigid upon PMMOF, possibly due to the incomplete imidization. Nevertheless, the *T_g_* of X-PI(370)/ZIF-8 remained unchanged regardless of the ZIF-8 loading, indicating no further chain rigidification. In contrast, the *T_g_* of the uncross-linked 6FDA-DAM/ZIF-8 MMMs increased slightly relative to that of 6FDA-DAM, indicating that the uncross-linked polymer became more rigid upon ZIF-8 incorporation (i.e., chain rigidification) (Figure S6a). Upon the in situ formation of ZIF-8 fillers, the uncross-linked polymer underwent more intensive rigidification than the cross-linked rigid polymer. As seen in Figure S6b, the X-PI(470)/ZIF-8 MMMs showed no distinct endothermic peaks in the DSC curves in the range of tested temperatures (up to 420 °C). Instead, the polymers were decomposed prior to the glass transition owing to their rigid structure (Figure S6b).

### 3.4. C_3_H_6_/C_3_H_8_ Separation Performance

**Figure 8:**
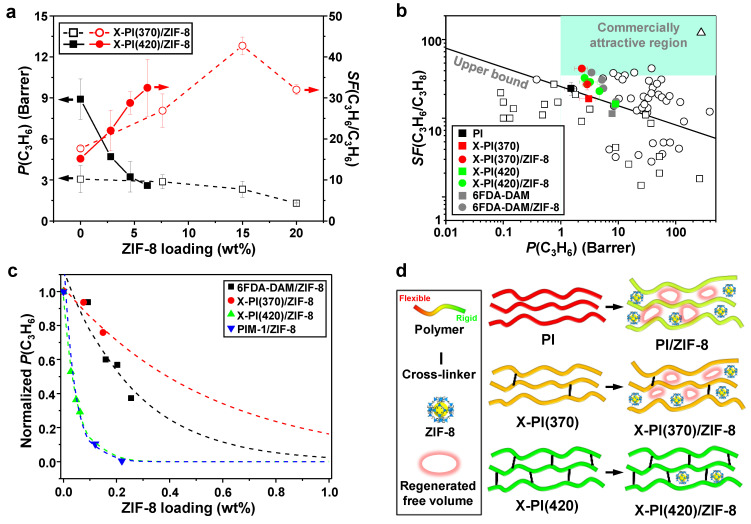
(**a**) C_3_H_6_ permeability (*P*) and C3 separation factor (*SF*) of the X-PI/ZIF-8 MMMs as a function of ZIF-8 loadings in MMMs; (**b**) upper bound plot of the C3 separation performances of the X-PI/ZIF-8 MMMs in comparison with those of the reported polymers (□) [36,37,38,39,40,41], MMMs (○) [14,16,42,43,44,45,46,47,48,49,50,51,52,53,54,55], and ZIF-8 (△) [42]; (**c**) normalized C_3_H_6_ permeabilities of in situ formed ZIF-8 containing MMMs as a function of ZIF-8 loadings; and (**d**) schematic illustrations of the free volume and chain flexibility changes before and after the PMMOF process. It is noted that the PI illustrated in (**d**) indicates 6FDA-DAM and that 6FDA-DAM:DABA (3:2) failed to produce PI/ZIF-8 MMMs (see Figure 4).

Figure 8a represents the C_3_H_6_ permeabilities and C3 separation factors of X-PI and X-PI/ZIF-8 MMMs as a function of ZIF-8 loading. Both of the X-PI membranes showed a higher C_3_H_6_ permeability and lower separation factor than the PI, which is unusual but consistent with the observation by Qiu at al. [23]. In general, polymer cross-linking leads to a reduction in polymer free volume, thereby decreasing the gas permeability. In contrast, cross-linking of the 6FDA-DAM:DABA polymer leads to an increase in the gas permeability due to the increased free volume by the nano-scale chain rearrangements, as studied in previous reports [23]. X-PI(420) showed a much higher C_3_H_6_ permeability than X-PI(370) with a slightly lower separation factor. This is likely due to the pronounced increase in the polymer-free volume upon cross-linking at a higher temperature. It is also expected that cross-linking provides a higher resistance of plasticization for the polymer phase for C_3_H_6_/C_3_H_8_ separation, mitigating the separation factor loss under high pressure [23].

Despite the lower ZIF-8 loadings, X-PI(420)/ZIF-8 MMMs showed a more dramatic decrease in C_3_H_6_ permeability than the X-PI(370)/ZIF-8 MMMs (Figure 8a and Table S2). Meanwhile, X-PI(420)/ZIF-8 showed a sharper increase in the separation factor than X-PI(370)/ZIF-8, likely attributed to the more severe polymer densification upon the in situ incorporation of ZIF-8 fillers in the free volume [14]. While both X-PI/ZIF-8 membranes showed a steady increase in the separation factor at the relatively low ZIF-8 fractions, X-PI(370)/ZIF-8 exhibited a sudden reduction in both the separation factor and C_3_H_6_ permeability when the loading was greater than ~15 wt% (Figure 8a). Based on this observation, it was assumed that the quality of X-PI(370)/ZIF-8 MMMs with ZIF-8 loadings less than 20 wt% was different from that of ~20 wt%. With respect to this, the result of the MMMs at ~20 wt% was excluded in the later discussion.

Both X-PI/ZIF-8 MMMs, in particular X-PI(370)/ZIF-8 MMMs, showed relatively high C3 separation factors with relatively low C_3_H_6_ permeability compared with those 6FDA-DAM-based MMMs reported by our group (Figure 8b). This is because of the lower C_3_H_6_ permeability of X-PI than 6FDA-DAM. The C3 separation performance of X-PI(420)/ZIF-8 tended to follow the upper bound with the increased filler loadings (Figure 8b). In contrast, X-PI(370)/ZIF-8 MMMs showed a significantly increased separation factor (i.e., from ~18 to ~43) with a relatively small reduction in permeability (i.e., from ~3.1 Barrer to ~2.3 Barrer), satisfying the criteria for commercial C3 separation (i.e., C_3_H_6_ permeability > 1 Barrer and C3 separation factor > 35) (Figure 8b) [34]. Although the X-PI(420)/ZIF-8 displayed a more pronounced permeability reduction than X-PI(370)/ZIF-8, X-PI(420)/ZIF-8 MMMs showed a higher C_3_H_6_ permeability than X-PI(370)/ZIF-8 MMMs at similar ZIF-8 loadings due to the higher permeability of the neat X-PI(420) polymers.

To further investigate the factors contributing to the permeability reduction upon PMMOF, polymers with different physical properties (i.e., free volume, chain rigidity, and swelling) were carefully selected and compared—X-PI(370) and X-PI(420), 6FDA-DAM [14], and PIM-1. The extents of the permeability reduction resulting from the in situ formation of ZIF-8 in different polymers was examined by normalizing the C_3_H_6_ permeabilities of MMMs by those of their neat polymer membranes as a function of ZIF-8 loading (Figure 8c). The slopes of the trend curves revealed that the permeability reduction was pronounced in the order of X-PI(370) < 6FDA-DAM << X-PI(420) < PIM-1 (Figure 8c). Initially, large polymer-free volumes appeared to have adverse effects on the permeability reduction. With the exceptionally large free volume of PIM-1, PIM-1/ZIF-8 exhibited the sharpest permeability reduction. Similarly, X-PI(420)/ZIF-8 MMMs showed a much more dramatic decrease in permeability than the X-PI(370)/ZIF-8 MMMs (note that X-PI(420) has a relatively larger free volume than X-PI(370)). Furthermore, despite the high chain rigidity of PIM-1 and X-PI(420) (*T_g_* of above 500 °C and 415 °C, respectively) [35], the most drastic permeability reduction of PIM-1/ZIF-8 and X-PI(420)/ZIF-8 MMMs indicated that chain rigidity might not be the dominant factor preventing the permeability decrease.

The considerable permeability reduction in PIM-1/ZIF-8 and X-PI(420)/ZIF-8 could be explained by the negligible polymer swellings and the absence of the subsequent chain rearrangements upon PMMOF. As there are no imide groups in PIM-1, the PIM-1/MMMs were prepared without hydrolysis and imidization steps (see Section 2). Both PIM-1 and X-PI(420) were less likely to be swollen during the PMMOF process, as PIM-1 did not go through the hydrolysis step and the fully cross-linked X-PI(420) was expected to show a high resistance to swelling. On the other hand, the less rigid X-PI(370)/ZIF-8 and the uncross-linked 6FDA-DAM/ZIF-8 MMMs showed much less permeability reduction. More swellable X-PI(370) and uncross-linked 6FDA-DAM were prone to generate additional free volumes upon the chain rearrangements (Figure 8d). As such, the regenerated free volume by swelling likely counteracted the densification by PMMOF, thereby mitigating the permeability decrease. Despite the lower degree of swelling of X-PI(370) due to its partial cross-linking compared with that of 6FDA-DAM, X-PI(370) prevented the permeability decrease more effectively than 6FDA-DAM (Figure 8c). This was likely attributed to the fact that the more rigid X-PI(370) prevented the further rigidification of polymers by fillers, likely mitigating permeability reduction. These observations strongly suggest that controlled cross-linking helps suppress the permeability reduction of MMMs prepared by PMMOF.

## 4. Conclusions

In conclusion, we demonstrated a strategy to mitigate the gas permeability reduction of ZIF-8-containing MMMs formed by the polymer-modification enabled in situ metal-organic framework formation (PMMOF). The strategy is based on controlled cross-linking of the continuous polymer phase (6FDA-DAM:DABA (3:2)). The properties of the polymer (i.e., free volume, chain mobility, swelling resistance, etc.) were dependent on the degree of cross-linking, consequently affecting the in situ formation of ZIF-8 filler particles. In the PMMOF process, more cross-linking led to much more reduced ZIF-8 loading. A substantially larger number of ZIF-8 particles were formed in the less cross-linked polymer (i.e., X-PI(370)) than in the more-cross-linked X-PI(420). Consequently, the X-PI(370)/ZIF-8 MMMs showed a significant C3 separation factor improvement (from ~18 to ~43 at 15wt% loading) with a minor C_3_H_6_ permeability reduction (from ~3.1 Barrer to ~2.3 Barrer), satisfying the commercially attractive C3 separation criteria. Furthermore, the permeability reduction of the MMMs prepared by PMMOF was investigated by comparing them with MMMs made of uncross-linked 6FDA-DAM and rigid PIM-1. The more swellable polymers (i.e., 6FDA-DAM and X-PI(370)) showed much less permeability reductions than the other less swellable polymers (i.e., PIM-1 and X-PI(420)). The X-PI(370)/ZIF-8 MMMs showed a much smaller permeability reduction than the 6FDA-DAM/ZIF-8 MMMs, suggesting that the rigidification by the PMMOF was mitigated in the X-PI(370). Two important factors were found to interplay in order to suppress permeability reduction in MMMs prepared by the PMMOF: (1) the polymer swelling upon PMMOF, which might regenerate free volume, and (2) the rigid polymer structures that are resistant to further rigidification upon PMMOF. Although the separation performance of X-PI(370)/ZIF-8 MMMs was mediocre compared with 6FDA-DAM/ZIF-8 MMMs due to the relatively lower permeance of X-PI(370) than 6FDA-DAM, we believe that the finding is significant as a possible strategy to prevent the permeability reduction of MMMs by PMMOF. Further optimization is necessary to achieve an improved C3 separation performance using cross-linked polymers.

## Supplementary Materials

The following supporting information can be downloaded at: https://www.mdpi.com/article/10.3390/membranes12100964/s1, Figure S1: Photographs of (a) PI, (b) X-PI(370), and (c) X-PI(420) in air and DMF for different times; Figure S2: XRD patterns of PI and two X-PIs coated on α-alumina supports that were cross linked under different conditions; Figure S3. Differential scanning calorimetric (DSC) thermogram of PI and two X-PIs, Figure S4. FT-IR spectra of X-PI(370), X-PAA(370), and X-PI(370)/ZIF-8 in comparison with neat PI, Figure S5. TGA thermogram of ZIF-8 and MMMs under air flow. The numbers in the parentheses are the ZIF-8 loadings, Figure S6. DSC thermograms of (a) 6FDA-DAM and (b) X-PI(420) and their MMMs with different ZIF-8 loadings, Table S1. Loading percentages of ZIF-8 in situ formed in cross-34 linked polymers, Table S2. Summary of binary (50/50) C3H6/C3H8 separation performances of polymer membranes and MMMs at ~1 atm and room temperature, Table S3. Comparison of relative physical properties of X-PI(370), X-PI(420), 6FDA-DAM, and PIM-1.

## References

[1] Robeson L.M. (2008). The upper bound revisited. J. Membr. Sci..

[2] Galizia M., Chi W.S., Smith Z.P., Merkel T.C., Baker R.W., Freeman B.D. (2017). 50th Anniversary Perspective: Polymers and Mixed Matrix Membranes for Gas and Vapor Separation: A Review and Prospective Opportunities. Macromolecules.

[3] Ebadi Amooghin A., Mashhadikhan S., Sanaeepur H., Moghadassi A., Matsuura T., Ramakrishna S. (2019). Substantial breakthroughs on function-led design of advanced materials used in mixed matrix membranes (MMMs): A new horizon for efficient CO_2_ separation. Prog. Mater. Sci..

[4] Wang Y., Wang X., Guan J., Yang L., Ren Y., Nasir N., Wu H., Chen Z., Jiang Z. (2019). 110th Anniversary: Mixed Matrix Membranes with Fillers of Intrinsic Nanopores for Gas Separation. Ind. Eng. Chem. Res..

[5] Song S.Q., Jiang H.F., Wu H., Zhao M.G., Guo Z.Y., Li B.Y., Ren Y.X., Wang Y.H., Ye C.M., Guiver M.D. (2022). Weakly pressure-dependent molecular sieving of propylene/propane mixtures through mixed matrix membrane with ZIF-8 direct-through channels. J. Membr. Sci..

[6] Sun Y.X., Tian L., Qiao Z.H., Geng C.X., Guo X.Y., Zhong C.L. (2022). Surface modification of bilayer structure on metal-organic frameworks towards mixed matrix membranes for efficient propylene/propane separation. J. Membr. Sci..

[7] Lee T.H., Jung J.G., Kim Y.J., Roh J.S., Yoon H.W., Ghanem B.S., Kim H.W., Cho Y.H., Pinnau I., Park H.B. (2021). Defect Engineering in Metal-Organic Frameworks Towards Advanced Mixed Matrix Membranes for Efficient Propylene/Propane Separation. Angew. Chem. Int. Ed..

[8] Hamid M.R.A., Jeong H.-K. (2018). Recent advances on mixed-matrix membranes for gas separation: Opportunities and engineering challenges. Korean J. Chem. Eng..

[9] Mahajan R., Koros W.J. (2002). Mixed matrix membrane materials with glassy polymers. Part 1. Polym. Eng. Sci..

[10] Dong G.X., Li H.Y., Chen V.K. (2013). Challenges and opportunities for mixed-matrix membranes for gas separation. J. Mater. Chem. A.

[11] Zhang C., Zhang K., Xu L., Labreche Y., Kraftschik B., Koros W.J. (2014). Highly scalable ZIF-based mixed-matrix hollow fiber membranes for advanced hydrocarbon separations. AIChE J..

[12] Marti A.M., Venna S.R., Roth E.A., Culp J.T., Hopkinson D.P. (2018). Simple Fabrication Method for Mixed Matrix Membranes with in Situ MOF Growth for Gas Separation. ACS Appl. Mater Interfaces.

[13] Ma L., Svec F., Lv Y., Tan T. (2019). In situ bottom–up growth of metal–organic frameworks in a crosslinked poly(ethylene oxide) layer with ultrahigh loading and superior uniform distribution. J. Mater. Chem. A.

[14] Park S., Abdul Hamid M.R., Jeong H.-K. (2019). Highly Propylene-Selective Mixed-Matrix Membranes by in Situ Metal–Organic Framework Formation Using a Polymer-Modification Strategy. ACS Appl. Mater. Interfaces.

[15] Park S., Cho K.Y., Jeong H.K. (2020). Polyimide/ZIF-7 mixed-matrix membranes: Understanding thein situconfined formation of the ZIF-7 phases inside a polymer and their effects on gas separations. J. Mater. Chem. A.

[16] Park S., Jeong H.-K. (2020). In-situ linker doping as an effective means to tune zeolitic-imidazolate framework-8 (ZIF-8) fillers in mixed-matrix membranes for propylene/propane separation. J. Membr. Sci..

[17] Zhang C., Lively R.P., Zhang K., Johnson J.R., Karvan O., Koros W.J. (2012). Unexpected Molecular Sieving Properties of Zeolitic Imidazolate Framework-8. J. Phys. Chem. Lett..

[18] Park S., Jeong H.K. (2020). Transforming polymer hollow fiber membrane modules to mixed-matrix hollow fiber membrane modules for propylene/propane separation. J. Membr. Sci..

[19] Ghosal K., Freeman B.D. (1994). Gas separation using polymer membranes: An overview. Polym. Adv. Technol..

[20] Monsalve-Bravo G.M., Dutta R.C., Bhatia S.K. (2020). Multiscale simulation of gas transport in mixed-matrix membranes with interfacial polymer rigidification. Microporous Mesoporous Mater..

[21] Chen C.C., Qiu W.L., Miller S.J., Koros W.J. (2011). Plasticization-resistant hollow fiber membranes for CO_2_/CH_4_ separation based on a thermally crosslinkable polyimide. J. Membr. Sci..

[22] Velioglu S., Ahunbay M.G., Tantekin-Ersolmaz S.B. (2016). Propylene/propane plasticization in polyimide membranes. J. Membr. Sci..

[23] Qiu W., Chen C.-C., Xu L., Cui L., Paul D.R., Koros W.J. (2011). Sub-Tg Cross-Linking of a Polyimide Membrane for Enhanced CO_2_ Plasticization Resistance for Natural Gas Separation. Macromolecules.

[24] Cui L.L., Qiu W.L., Paul D.R., Koros W.J. (2011). Responses of 6FDA-based polyimide thin membranes to CO_2_ exposure and physical aging as monitored by gas permeability. Polymer.

[25] Hillock A.M.W., Koros W.J. (2007). Cross-linkable polyimide membrane for natural gas purification and carbon dioxide plasticization reduction. Macromolecules.

[26] Qiu W.L., Xu L.R., Chen C.C., Paul D.R., Koros W.J. (2013). Gas separation performance of 6FDA-based polyimides with different chemical structures. Polymer.

[27] Kwon H.T., Jeong H.-K. (2013). Highly propylene-selective supported zeolite-imidazolate framework (ZIF-8) membranes synthesized by rapid microwave-assisted seeding and secondary growth. Chem. Commun..

[28] Yin H., Kim H., Choi J., Yip A.C.K. (2015). Thermal stability of ZIF-8 under oxidative and inert environments: A practical perspective on using ZIF-8 as a catalyst support. Chem. Eng. J..

[29] Lively R.P., Dose M.E., Xu L.R., Vaughn J.T., Johnson J.R., Thompson J.A., Zhang K., Lydon M.E., Lee J.S., Liu L. (2012). A high-flux polyimide hollow fiber membrane to minimize footprint and energy penalty for CO_2_ recovery from flue gas. J. Membr. Sci..

[30] Eguchi H., Kim D.J., Koros W.J. (2015). Chemically cross-linkable polyimide membranes for improved transport plasticization resistance for natural gas separation. Polymer.

[31] Kratochvil A.M., Koros W.J. (2008). Decarboxylation-Induced Cross-Linking of a Polyimide for Enhanced CO_2_ Plasticization Resistance. Macromolecules.

[32] Tarleton E.S., Robinson J.P., Salman M. (2006). Solvent-induced swelling of membranes—Measurements and influence in nanofiltration. J. Membr. Sci..

[33] Mahkam M., Doostie L. (2005). The relation between swelling properties and cross-linking of hydrogels designed for colon-specific drug delivery. Drug Deliv..

[34] Craig Colling G.H., Bartels J. (2002). Processes using solid perm-selective membranes in multiple groups for simultaneous recovery of specified products from a fluid mixture. U.S. Patent.

[35] Du N.Y., Robertson G.P., Pinnau I., Guiver M.D. (2010). Polymers of Intrinsic Microporosity with Dinaphthyl and Thianthrene Segments. Macromolecules.

[36] Krol J.J., Boerrigter M., Koops G.H. (2001). Polyimide hollow fiber gas separation membranes: Preparation and the suppression of plasticization in propane/propylene environments. J. Membr. Sci..

[37] Bai S., Sridhar S., Khan A.A. (1998). Metal-ion mediated separation of propylene from propane using PPO membranes. J. Membr. Sci..

[38] Sridhar S., Khan A.A. (1999). Simulation studies for the separation of propylene and propane by ethylcellulose membrane. J. Membr. Sci..

[39] Staudt-Bickel C., Koros W.J. (2000). Olefin/paraffin gas separations with 6FDA-based polyimide membranes. J. Membr. Sci..

[40] Chng M.L., Xiao Y.C., Chung T.S., Toriida M., Tamai S. (2009). Enhanced propylene/propane separation by carbonaceous membrane derived from poly (aryl ether ketone)/2,6-bis(4-azidobenzylidene)-4-methyl-cyclohexanone interpenetrating network. Carbon.

[41] Xiao Y.C., Chung T.S. (2011). Grafting thermally labile molecules on cross-linkable polyimide to design membrane materials for natural gas purification and CO_2_ capture. Energy Environ. Sci..

[42] Zhang C., Dai Y., Johnson J.R., Karvan O., Koros W.J. (2012). High performance ZIF-8/6FDA-DAM mixed matrix membrane for propylene/propane separations. J. Membr. Sci..

[43] Sun H.X., Ma C., Wang T., Xu Y.Y., Yuan B.B., Li P., Kong Y. (2014). Preparation and Characterization of C60-Filled Ethyl Cellulose Mixed-Matrix Membranes for Gas Separation of Propylene/Propane. Chem. Eng. Technol..

[44] Liu Y., Chen Z.J., Liu G.P., Belmabkhout Y., Adil K., Eddaoudi M., Koros W. (2019). Conformation-Controlled Molecular Sieving Effects for Membrane-Based Propylene/Propane Separation. Adv. Mater..

[45] Ma X.H., Swaidan R.J., Wang Y.G., Hsiung C.E., Han Y., Pinnau I. (2018). Highly Compatible Hydroxyl-Functionalized Microporous Polyimide-ZIF-8 Mixed Matrix Membranes for Energy Efficient Propylene/Propane Separation. ACS Appl. Nano Mater..

[46] Yu J., Wang C., Xiang L., Xu Y., Pan Y. (2018). Enhanced C3H6/C3H8 separation performance in poly(vinyl acetate) membrane blended with ZIF-8 nanocrystals. Chem. Eng. Sci..

[47] Liu D.H., Xiang L., Chang H., Chen K., Wang C.Q., Pan Y.C., Li Y.S., Jiang Z.Y. (2019). Rational matching between MOFs and polymers in mixed matrix membranes for propylene/propane separation. Chem. Eng. Sci..

[48] Chi W.S., Kim S.J., Lee S.J., Bae Y.S., Kim J.H. (2015). Enhanced Performance of Mixed-Matrix Membranes through a Graft Copolymer-Directed Interface and Interaction Tuning Approach. ChemSusChem.

[49] Japip S., Wang H., Xiao Y.C., Chung T.S. (2014). Highly permeable zeolitic imidazolate framework (ZIF)-71 nano-particles enhanced polyimide membranes for gas separation. J. Membr. Sci..

[50] An H., Park S., Kwon H.T., Jeong H.K., Lee J.S. (2017). A new superior competitor for exceptional propylene/propane separations: ZIF-67 containing mixed matrix membranes. J. Membr. Sci..

[51] Oh J.W., Cho K.Y., Kan M.Y., Yu H.J., Kang D.Y., Lee J.S. (2020). High-flux mixed matrix membranes containing bimetallic zeolitic imidazole framework-8 for C3H6/C3H8 separation. J. Membr. Sci..

[52] Shen Q., Cong S.Z., He R.R., Wang Z., Jin Y.H., Li H., Cao X.Z., Wang J., Van der Bruggen B., Zhang Y.T. (2019). SIFSIX-3-Zn/PIM-1 mixed matrix membranes with enhanced permeability for propylene/propane separation. J. Membr. Sci..

[53] Askari M., Chung T.S. (2013). Natural gas purification and olefin/paraffin separation using thermal cross-linkable co-polyimide/ZIF-8 mixed matrix membranes. J. Membr. Sci..

[54] Zhang Q., Li H.B., Chen S., Duan J.G., Jin W.Q. (2020). Mixed-matrix membranes with soluble porous organic molecular cage for highly efficient C3H6/C3H8 separation. J. Membr. Sci..

[55] Lin R.J., Ge L., Diao H., Rudolph V., Zhu Z.H. (2016). Propylene/propane selective mixed matrix membranes with grape-branched MOF/CNT filler. J. Mater. Chem. A.

