# Supplementary material for: Cross-Linked Polyimide/ZIF-8 Mixed-Matrix Membranes by In Situ Formation of ZIF-8: Effect of Cross-Linking on Their Propylene/Propane Separation
Source: Membranes (Basel). 2022 Oct 1;12(10):964. doi: 10.3390/membranes12100964 (PMC9609502; doi:10.3390/membranes12100964)
Supplement: Supplementary file 1 [file membranes-12-00964-s001.zip › membranes-1922904-supplementary.pdf]

Supporting Information

Cross-Linked Polyimide/ZIF-8 Mixed-Matrix Membranes by In Situ Formation of ZIF-8: Effect of Cross-Linking on Their Propylene/Propane Separation

Sunghwan Park [1,2,*] and Hae-Kwon Jeong [3,4,*]

[1] School of Energy Materials & Chemical Engineering, Kyungpook National University, Sangju-si 37224, Republic of Korea
[2] Department of Advanced Science and Technology Convergence, Kyungpook National University, Sangju-si 37224, Republic of Korea
[3] Artie McFerrin Department of Chemical Engineering, Texas A&M University, 3122 TAMU, College Station, TX 77843-3122, USA
[4] Department of Materials Science and Engineering, Texas A&M University, 3122 TAMU, College Station, TX 77843-3122, USA
* Correspondence: sunghwan@knu.ac.kr (S.P.); hjeong7@tamu.edu (H.-K.J.); Tel.: +82-54-530-1331 (S.P.); +1-979-862-4850 (H.-K.J.); Fax: +1-979-845-6446 (H.-K.J.)

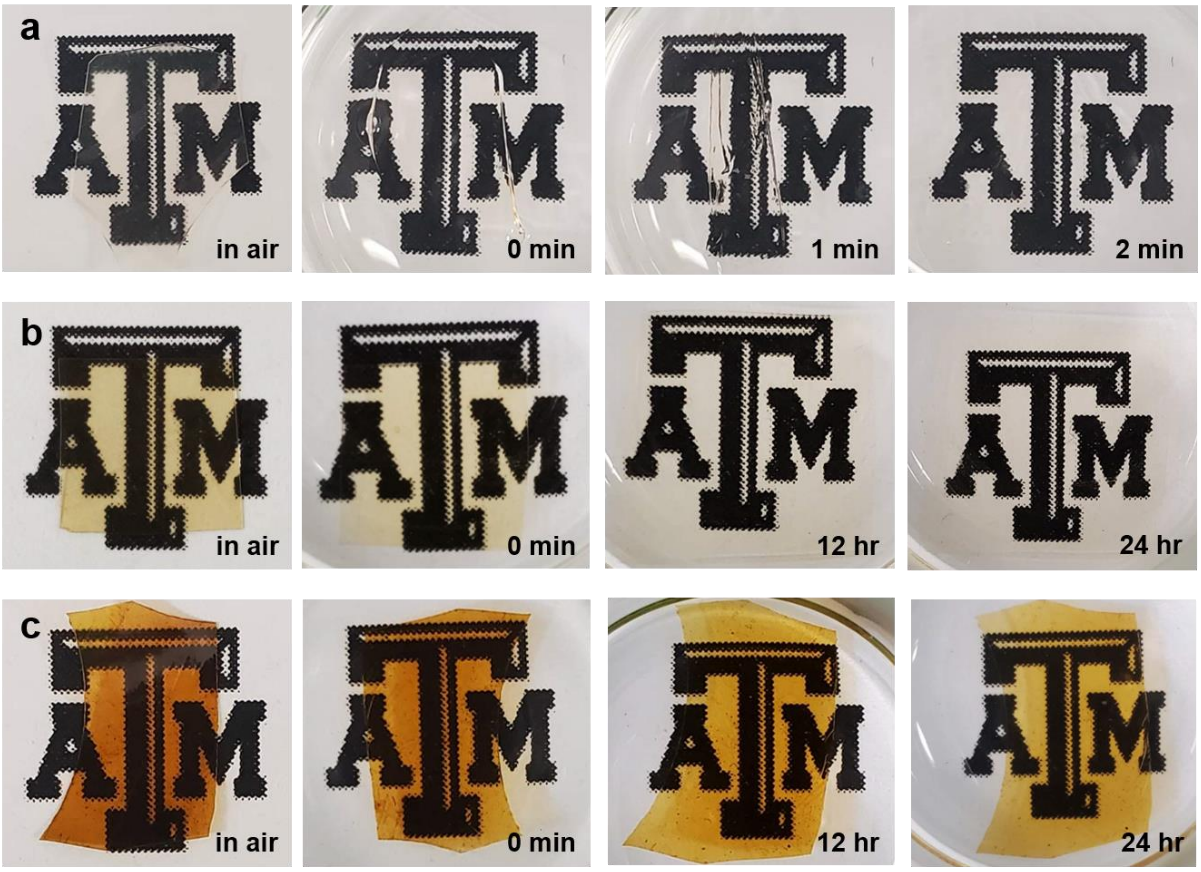

Figure S1. Photographs of (a) PI, (b) X-PI(370), and (c) X-PI(420) in air and DMF for different times.

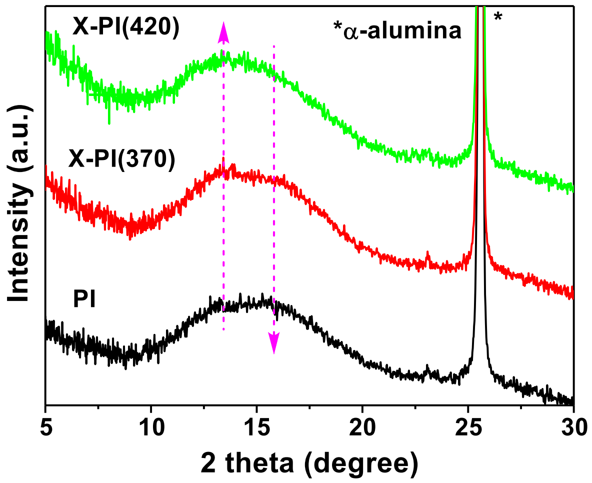

Figure S2. XRD patterns of PI and two X-PIs coated on α-alumina supports that were cross-linked under different conditions.

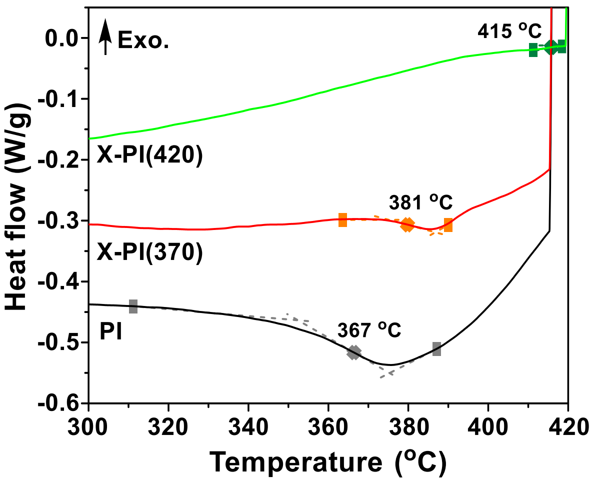

Figure S3. Differential scanning calorimetric (DSC) thermogram of PI and two X-PIs.

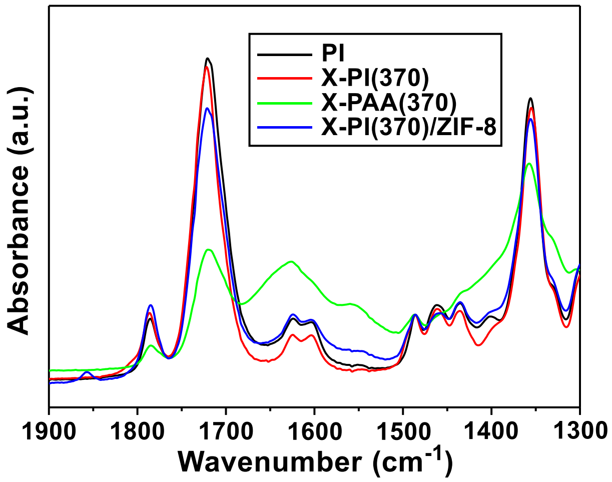

Figure S4. FT-IR spectra of X-PI(370), X-PAA(370), and X-PI(370)/ZIF-8 in comparison with neat PI.

Table S1. Loading percentages of ZIF-8 *in-situ* formed in cross-linked polymers.

Sample	Zinc concentration in ion exchange solution		
	0.5 M	1.0 M	1.5 M
X-PI(370)/ZIF-8	7.6 ± 1.9 wt%	14.9 ± 3.3 wt%	19.7 ± 2.8 wt%
X-PI(420)/ZIF-8	2.8 ± 1.0 wt%	4.8 ± 0.8 wt%	6.2 ± 1.7 wt%

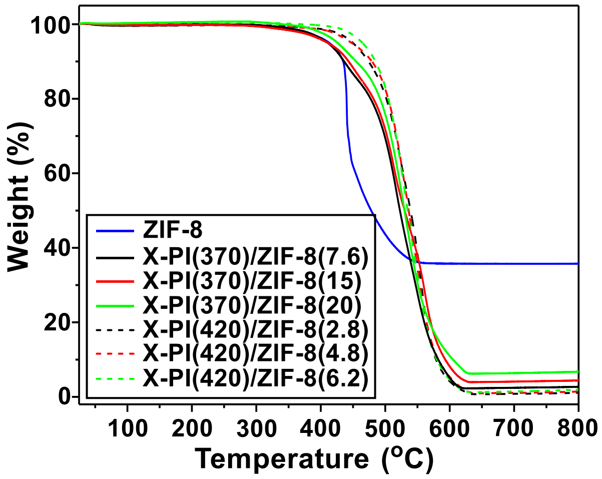

Figure S5. TGA thermogram of ZIF-8 and MMMs under air flow. The numbers in the parentheses are the ZIF-8 loadings.

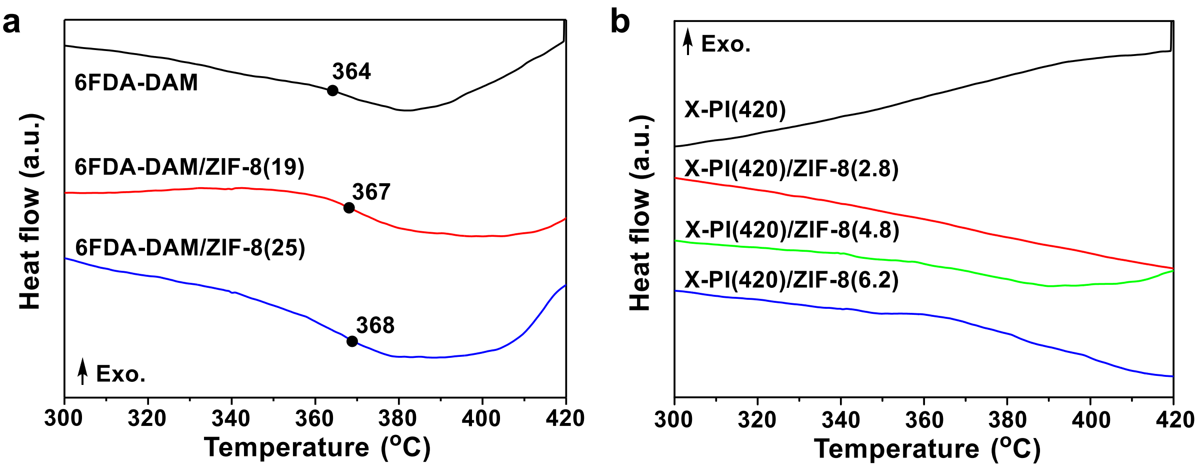

Figure S6. DSC thermograms of (a) 6FDA-DAM and (b) X-PI(420) and their MMMs with different ZIF-8 loadings.

Table S2. Summary of binary (50/50) C_3H_6/C_3H_8 separation performances of polymer membranes and MMMs at ~ 1 atm and room temperature.

Sample	ZIF-8 loading (wt%)	C_3H_6 permeability (Barrer)	C_3H_6/C_3H_8 separation factor
PI	-	1.51 ± 0.34	23.9 ± 4.4
X-PI(370)	-	3.06 ± 0.98	17.7 ± 0.6
X-PI(370)/ZIF-8	7.6	2.87 ± 0.51	26.9 ± 4.1
	14.9	2.32 ± 0.58	42.7 ± 2.1
	19.7	1.30 ± 0.04	32.0 ±1.2
X-PI(420)	-	8.91 ± 1.48	15.2 ± 0.7
X-PI(420)/ZIF-8	2.8	4.71 ± 0.01	22.0 ± 5.0
	4.8	3.23 ± 0.08	28.8 ± 2.7
	6.2	2.59 ± 0.15	32.5 ± 6.9

Table S3. Comparison of relative physical properties of X-PI(370), X-PI(420), 6FDA-DAM, and PIM-1.

Polymer	Free volume	Chain rigidity[b]	Degree of swelling
X-PI(370)	Low	Moderate	High
X-PI(420)	Moderate	High	Low
6FDA-DAM	High	Moderate	High
PIM-1	Very high	Very high	Low

[a] The free volumes were evaluated based on intrinsic gas permeabilities. [b] The chain rigidities were estimated by T_gs determined by DSC curves.
